# GaN Vertical Transistors with Staircase Channels for High-Voltage Applications

**DOI:** 10.3390/ma16020582

**Published:** 2023-01-06

**Authors:** Kuntal Barman, Dai-Jie Lin, Rohit Gupta, Chih-Kang Chang, Jian-Jang Huang

**Affiliations:** 1 Graduate Institute of Photonics and Optoelectronics, National Taiwan University, Taipei 10617, Taiwan; 2 Department of Electrical Engineering, National Taiwan University, Taipei 10617, Taiwan

**Keywords:** vertical transistor, vertical GaN transistor, high power transistor, GaN-based high power transistor, staircase channel GaN transistor, GaN on GaN technology

## Abstract

In this study, we propose and simulate the design of a non-regrowth staircase channel GaN vertical trench transistor, demonstrating an exceptional threshold and breakdown characteristic for high power and high frequency applications. The unique staircase design provides a variable capacitance through the gate-dielectric-semiconductor interface, which results in a high breakdown voltage of 1.52 kV and maintains a channel on-resistance of 2.61 mΩ∙cm^2^. Because of the variable length and doping profile in the channel region, this model offers greater flexibility to meet a wide range of device application requirements.

## 1. Introduction

Current telecommunications rely on the availability of power supplies. With the explosive demand for high bandwidth and 24/7 connectivity, the electrical infrastructure is facing the challenges of increasing the power conversion efficiency. In recent years, wide bandgap (WBG) materials and their corresponding power devices are attracting much attention. Gallium Nitride (GaN) is a critical WBG material for high-power, high-speed electronics. Compared to other mature devices based on Si or GaAs, GaN-based transistors exhibit exceptional properties, such as low specific on-resistance (R_on_), high breakdown-voltage (V_br_), and high operating frequency, giving a fixed device geometry [1,2,3,4,5]. Lateral GaN transistors on Si substrate have been widely studied. They are currently adopted in commercial power electronics due to their cost-effectiveness, as compared with GaN-based devices grown on GaN, SiC or other substrates. However, lateral transistors have two main limitations. First, to sustain high voltage, a large lateral gate to drain spacing is required [6], which inevitably increases the chip size. Second, thermal energy is generated and distributed primarily in the top epilayers because they are where current flow occurs [7,8], making the corresponding thermal resistance per unit chip area higher than vertical type transistors. Therefore, similar to Si power MOSFETs (metal-oxide semiconductor field-effect transistors), GaN vertical transistors have been studied by growing GaN epilayers on GaN substrate. Because lateral GaN power devices on Si substrate suffer from current collapse during high-voltage operation due to carrier trapping in the buffer region [9,10]. GaN on GaN devices have additional advantages. They avoid buffer-induced carrier trappings, making operating voltage much higher than 600 V feasible.

Several works have been reported in the last few years to develop GaN-based vertical transistors [11,12,13]. Based on the device architecture, there are primarily two types of structures [14,15]. The first one is the current aperture vertical electron transistor (CAVET) [16,17], consisting of a 2DEG channel induced by polarization in the AlGaN/GaN interface. In contrast, the second is the vertical GaN trench MOSFET [18,19], which is operated based on the inversion layer. Since the fundamental vertical structure consists of a vertical p-n junction, material regrowth is crucial for most of the above vertical transistors. The pattern etching followed by the regrowth process induces impurities in the interface [20]. Although a post thermal annealing and the in-situ cleaning process can neutralize most impurities [21,22], the regrowth limits the device performance and production yield. For example, the breakdown performance is limited to around 600 V for the trench CAVET [23] and the regrowth-based MOSFET [24]. On the other hand, for a GaN vertical fin-power transistor [25,26] in which material regrowth isn’t required, a higher V_br_ (>1kV) is demonstrated.

In this work, we propose and simulate a novel staircase trench design that can attain higher V_br_ and low R_on_. The design is based on an n^+^-i-P-n-n^+^ non-regrowth layer structure with a unique staircase-like channel region. The staircase side wall can be realized with a non-regrowth process, which is one of the essential criteria for minimizing interface defects.

## 2. Design and Simulation

**Figure 1:**
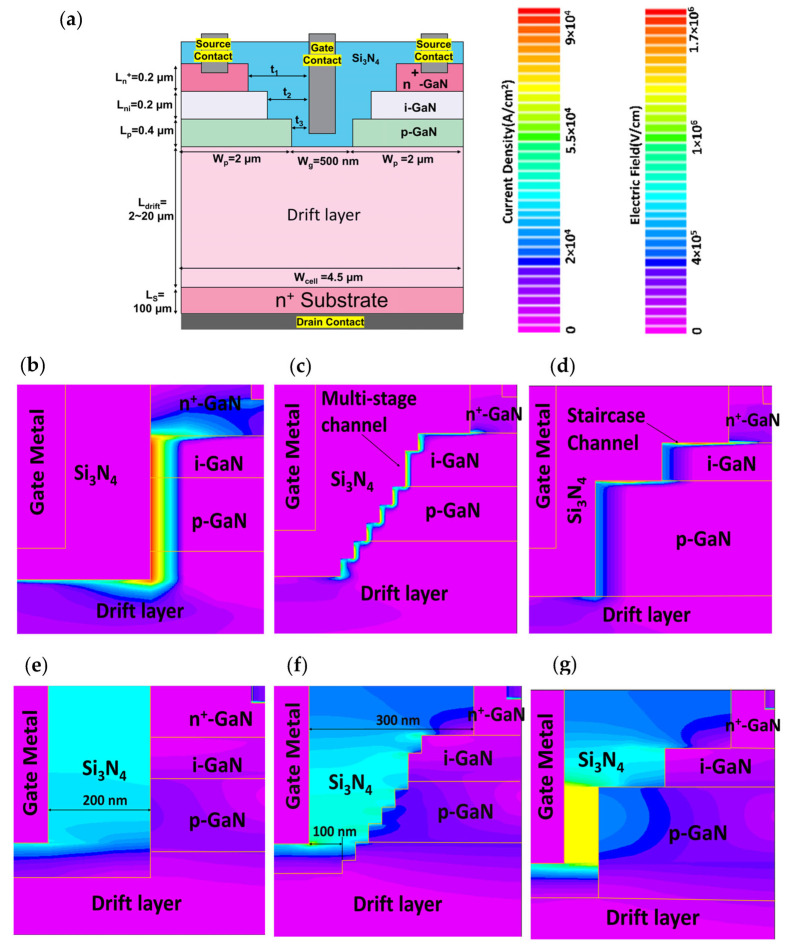
(**a**) Device structure of the proposed staircase channel transistor. Comparison of current density distributions in traditional straight channel (**b**), multi-stage staircase channel (**c**), and staircase channel (**d**) transistors. (**e**–**g**) Comparison of Electric field profiles of the traditional straight channel, multi-stage staircase channel, and staircase channel transistors respectively.

In order to obtain an in-depth study of the device, a systematic approach to performance analysis is carried out. The model is simulated based on standard semiconductor device physics using Poisson’s equation, carrier continuity equation, and transport equation. Moreover, the breakdown characteristic is calculated by considering Selberherr’s impact ionization model [27,28,29]. The staircase device structure is shown in Figure 1a. In practice, the fabrication of a staircase channel device can be realized by first performing three ICP-RIE (Inductively Coupled Plasma–Reactive Ion Etching) steps; each stops at i-GaN, p-GaN, and n-type drift layer. The etched thickness (though there is tolerance of an over-etch of each step) can be precisely monitored with plasma end-point detection during ICP-RIE etching. Multi-dielectric layer coating with a total thickness higher than 900 nm is proposed to fill up the staircase trench followed by source contact VIA hole etching and source electrode deposition. The gate electrode can be then deposited after the gate VIA hole definition. The drain contact is coated after the drift layer is grinded to the desired thickness. Multiple etching steps increase surface roughness and induce defects which may degrade the carrier mobility. Such surface defects can be effectively passivated with a proper recipe of GaN etching and dielectric deposition. Additionally, some advanced etching methods, such as digital etching with N_2_ plasma treatment, can minimize exposed defects [30]. Parametric analysis is performed based on two major parameters: device geometry and doping concentrations. As shown in Figure 1, the model is studied by varying the drift length (L_drift_) of the drift layer between 2 and 20 µm and drift doping concentration (n_drift_) between 5 × 10^15^ and 5 × 10^16^/cm^3^. The gate to n^+^-GaN, i-GaN, and p-GaN length, t_1_, t_2_, t_3,_ is 300, 200, and 100 nm, respectively. The typical value of p-GaN doping concentration (n_p_), source contact n^+^-GaN doping concentration (n^+^), and n_drift_ is kept at 2 × 10^17^, 8 × 10^18^, and 1 × 10^16^ cm^−3^, respectively. To highlight the dependence of electrical properties on the device profile near the gate-source region, we compared the proposed staircase structure with the traditional straight channel trench gate (see Figure 1b) and multi-stage staircase (see Figure 1c) structures. The purpose of the multi-stage staircase profile is to understand the electrical field and current distribution when multiple-step etching is applied to the staircase device. It can be considered as a tapered structure if the number of staircase steps is infinite. The gate to channel distance is 200 nm for a straight channel device while that of a multi-stage channel device decreases from 300 to 100 nm as labeled in Figure 1e,f. The epi-structures of all three types of devices are the same throughout the study.

The on-state channel current density of the traditional straight channel trench gate, multi-stage staircase, and staircase channel device are plotted in Figure 1b–d, respectively. The above channel formation is investigated by applying a gate to source voltage higher than the threshold voltage (V_th_) (7 V in this work). Among the devices, current density along the vertical sidewall of the traditional straight channel trench gate (Figure 1b) is the highest and the most uniform, with the current distribution deep into the sidewall. For a multi-stage device (Figure 1c), the current density increases with the depth of the channel due to the gradually reduced distance from gate metal. As for the staircase channel, current density depends on the corresponding gate-channel dielectric spacing (e.g., t_1_, t_2_, t_3_) of each step (see Figure 1d). For instance, because of a smaller dielectric thickness between the gate metal and p-GaN, t_3_, a stronger electric field in the channel leads to narrower carrier confinement, thus a better gate bias control and a higher channel transconductance.

Comparative electric field distribution of the devices is shown in Figure 1e–g. For the staircase design (shown in Figure 1g), due to the shortest spacing between the gate and p-GaN region, the electric field in the p-GaN layer is the strongest. It creates a strong inversion at the interface of the p-GaN region for the staircase channel. It is observed that the electric field at the dielectric/semiconductor interface is strong and exponentially decays away from the interface. The e-field gradient is lower in the intrinsic region than in the p-GaN region, which is one of the reasons for a broader carrier distribution in the intrinsic region.

**Figure 2:**
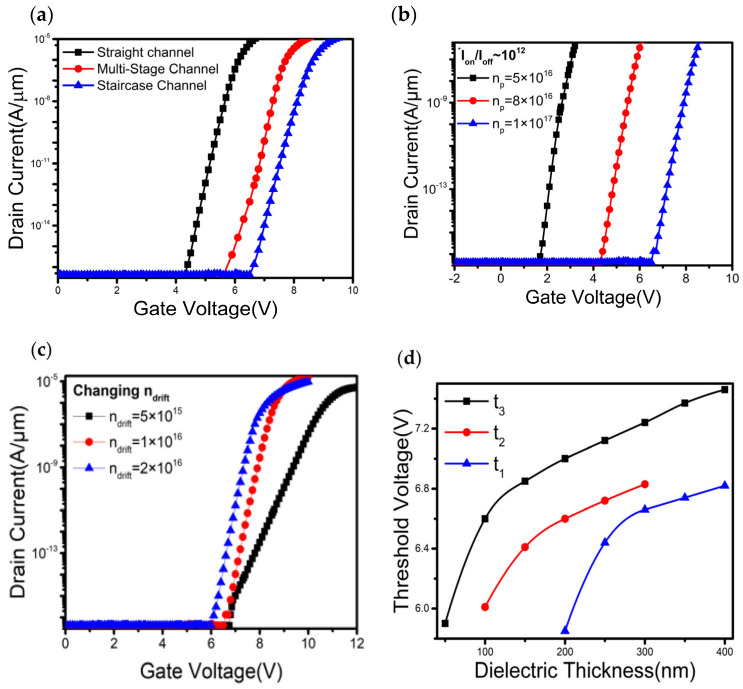
Transfer characteristics of the devices (**a**) Transfer curves of different types of vertical transistors with the same layer thickness and doping concentration. (**b**) Transfer curves of the staircase channel transistor at various n_p_. (**c**) Variation of transfer curves of the staircase channel transistor with n_drift_. For (**a**–**c**), the drain voltage for demonstrating transfer curves is 5V. (**d**) Variations of threshold voltage to the lateral dielectric layer (staircase) thickness.

V_th_ is one of the key parameters to understanding the device’s switching and leakage performance. A greater value of V_th_ (generally greater than 3 V) is always expected in high-power applications. Conceptually, it is mainly determined by the p-type and n-type drift layers. The comparative transfer characteristics between straight, multistage, and staircase channels are shown in Figure 2a, in which staircase structure exhibits a higher threshold voltage than the other two types of transistors. For the staircase channel transistor, the variation of V_th_ with respect to different values of n_p_ is plotted in Figure 2b. It is observed that the V_th_ significantly increases with n_p_. Since a higher n_p_ has a stronger depletion and reverse field, it requires a larger gate to source potential (V_gs_) to accumulate carriers at the dielectric-semiconductor interface and to switch on the device. Hence a large V_th_, extracted from linear extrapolation of the transfer curve, around 6.6 V can be achieved at n_p_ of 1 × 10^17^ cm^−3^ while keeping n_drift_, L_p_, L_drift_ at 1 × 10^16^ cm^−3^, 200 nm, and 12 µm, respectively. Figure 2c also shows a slight dependence of V_th_ on the n_drift_. Variations of V_th_ to t_1_, t_2_, and t_3_ of the staircase device are explored in Figure 2d. The thickness of a certain parameter is varied by keeping the rest two to their original assigned values. V_th_ is calculated using linear extrapolation of the transfer curves. It demonstrates that V_th_ increases with layer thickness. 

However, the threshold voltage predominantly depends on t_3_.

**Figure 3:**
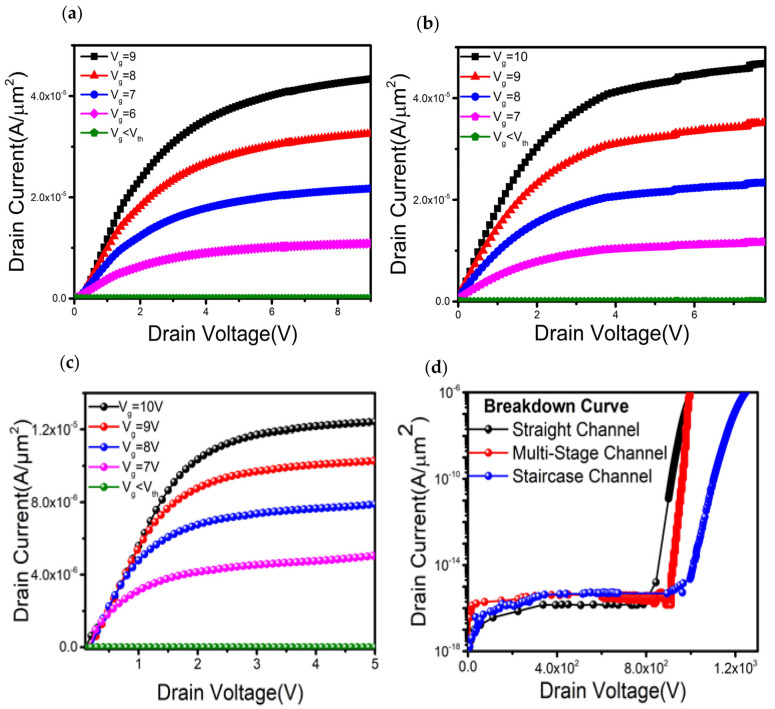
(**a**) Output characteristics of the straight channel trench channel transistor. (**b**) Output characteristics of the multi-stage channel transistor. (**c**) Output characteristics of the staircase channel transistor. (**d**) Comparisons of breakdown characteristics of the straight channel trench gate, multi-stage staircase and staircase channel transistors, where the layer thickness and corresponding doping concentration are kept the same for all structures.

The output characteristics were obtained by sweeping the drain voltage with fixed gate voltages. Figure 3a–c represent the output characteristics of straight, multi-stage, and staircase channels, respectively. Sharp increase of linear current reflects very low R_on_ values of 1.54 mΩ∙cm^2^, 1.63 mΩ∙cm^2^, and 1.77 mΩ∙cm^2^ for the straight, multistage, and staircase devices, respectively. For this study, the thickness and doping concentration of each layer were kept constant throughout all types of devices. The breakdown characteristics are obtained by sweeping the drain to source voltage and keeping the V_gs_ less than V_th_. A comparative breakdown analysis was carried out among the devices and is plotted in Figure 3d, where the L_drift_ is 9 µm and all other structural parameters were kept the same among devices. V_br_, defined as the voltage at which the reverse current is 10^−10^ A/µm^2^, of the traditional straight channel trench, multi- stage staircase, and staircase channel transistor is 880, 972, and 1086 V, respectively. It is shown that the staircase design can have superior breakdown performance compared to the other two types of vertical design. This phenomenon can be explained by considering the channel current density depicted in Figure 1b. Moreover, with a careful design of L_drift_ (~20 µm), a large V_br_ around 2.12 kV can be achieved while keeping n_p_, n_drift_, and L_p_ at 2 × 10^17^ cm^−3^, 1 × 10^16^ cm^−3^, and 400 nm, respectively.

**Figure 4:**
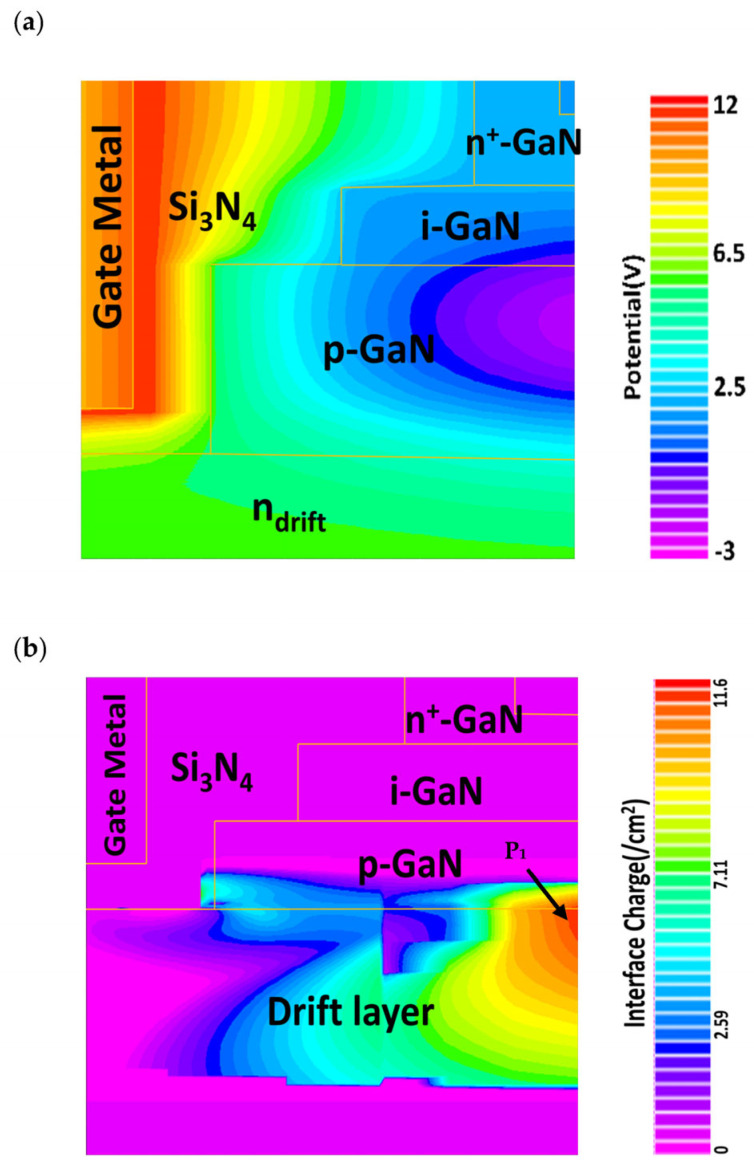
(**a**) Variation of channel potential of the staircase channel transistor.; (**b**) Generation rate of charge carriers by impact ionization.

Conceptually, a larger L_drift_ with a smaller n_drift_ can help distribute the field generated by large drain voltage so that the effective field is lower than the critical field for avalanche breakdown. Therefore, parameter optimization is crucial to satisfy different application requirements. The variation of the staircase channel potential is depicted in Figure 4a. It is observed that the channel potential at dielectric/semiconductor interface is strong and exponentially decays away from the interface. The channel potential gradient is lower in the intrinsic region than that in the p-GaN region, which is one of the reasons for a broader carrier distribution in the intrinsic region. The breakdown occurs as a result of an avalanche process triggered by impact ionization. At a very high drain to source potential, electrons at p-GaN and drift n-GaN interface obtain high kinetic energy and undergo scattering events, where the excess energy transferred to neighboring atom, generating new electron-hole pairs. This process continues and rapid carrier multiplication occurs. Sudden surge in generated carriers results a direct current flow between n^+^ source and drain terminal, hence the gate loses its control over the channel and avalanche breakdown occurs. The generation rate of interface charges by impact ionization is represented in Figure 4b. It is observed that the impact ionization rate is maximum at the edges as indicated as point P_1_.

**Figure 5:**
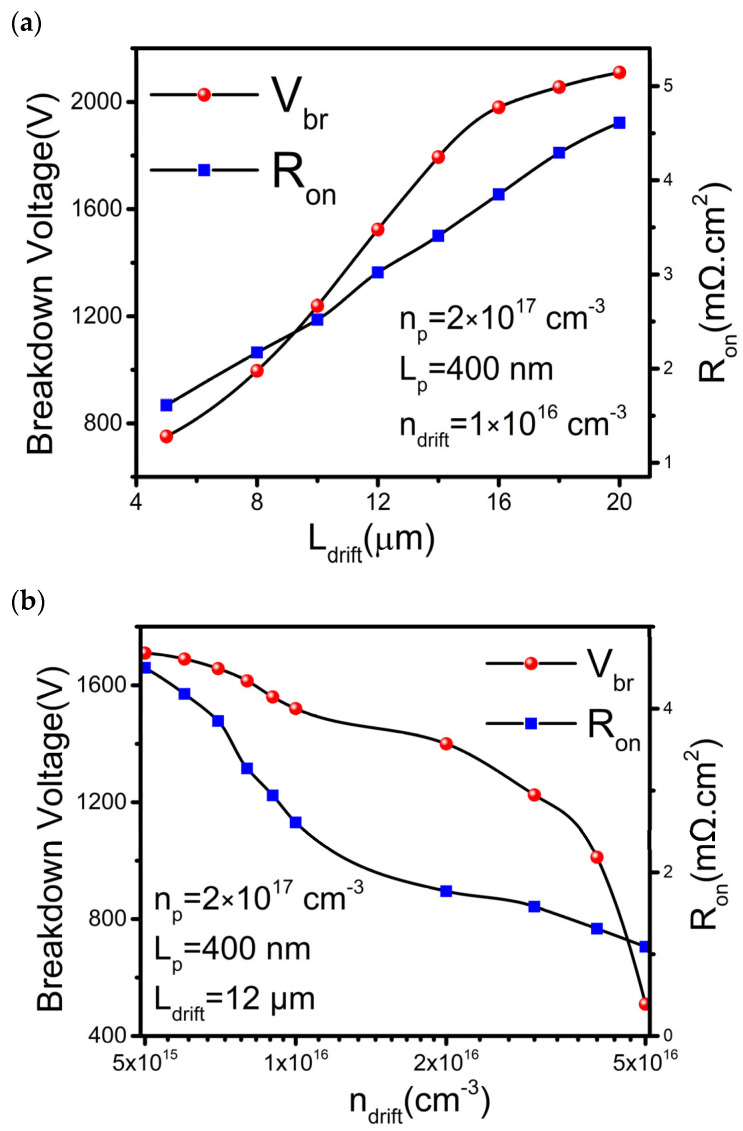
Optimization of V_br_ with various parameters: (**a**) drift length (L_drift_), (**b**) Drift doping concentration (n_drift_), with constant n_p_, L_p_ and L_drift_ at 2 × 10^17^ cm^−3^, 400 nm and 12 µm respectively.

In general, the optimization is performed based on the desired application. In this study, optimization is carried out to achieve balanced performance between high V_br_ and low R_on_. First, the L_drift_ is optimized while maintaining other parameters, n_p_, L_p_, and n_drift_, constant at 2 × 10^17^ cm^−3^, 400 nm, and 1 × 10^16^ cm^−3^, respectively. The L_drift_ varies from 2 to 20 µm. As shown in Figure 5a, the V_br_ increases with L_drift_ but gradually saturated when L_drift_ exceeds 16 µm. On the other hand, the R_on_ increases linearly with L_drift_. By setting L_drift_ to be 12 µm, we can obtain a R_on_ of 2.61 mΩ∙cm^2^ and a V_br_ of 1.52 kV. Next, the n_drift_ is optimized by keeping L_drift_ at 12 µm, as plotted in Figure 5b. By increasing n_drift_, the V_br_ is reduced. The result is due to the reduced R_on_ when the carrier density between the source and drain increases. When the n_drift_ is larger than 1 × 10^16^ cm^−3^, a sharp decrease of V_br_ but a slow decrease of R_on_ is observed. Hence, the n_drift_ around 1 × 10^16^ cm^−3^ or lower is most acceptable for high-power applications.

**Figure 6:**
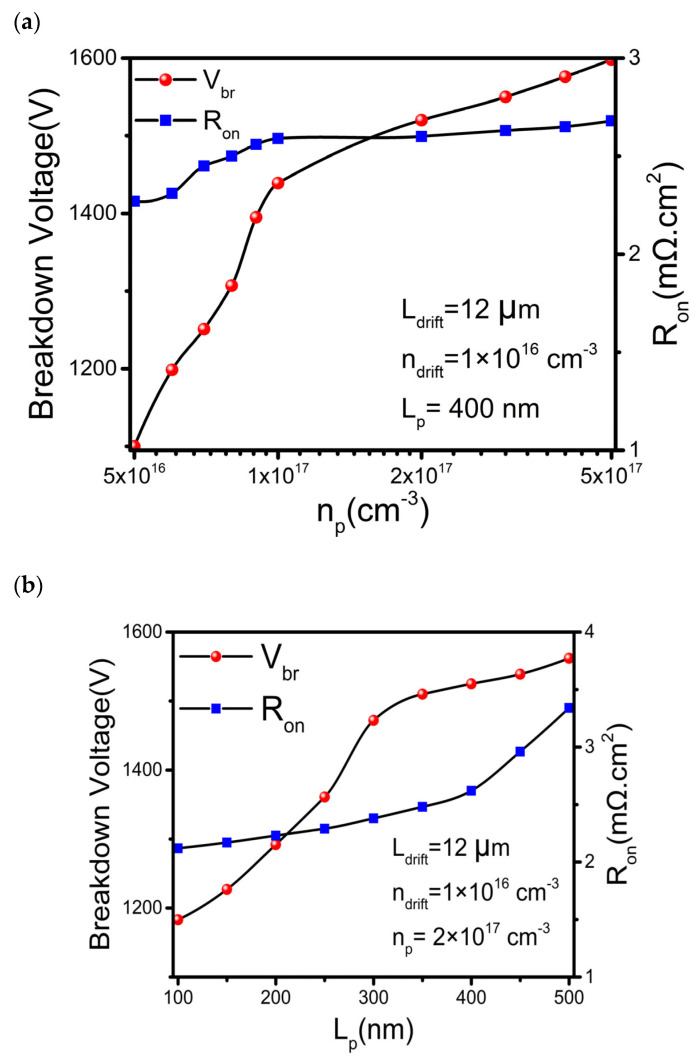
Optimization of V_br_ with respect to (**a**) p-doping concentration (n_p_) and (**b**) P-layer thickness (Lp).

Moreover, the p-type region is vital for switching performance and breakdown characteristics. Optimization is carried out by correlating n_p_ to L_p_. Generally, a thicker p-layer with a higher doping concentration leads to an increase of channel resistance. It improves the breakdown voltage but the channel resistance increase slows down the switching operation. As plotted in Figure 6a, when n_p_ increases, the V_br_ increases linearly and saturates at n_p_ around 2 × 10^17^ cm^−3^. Similarly, the R_on_ increases slowly until n_p_ reaches 1 × 10^17^ cm^−3^. In such a case, the optimized np is around 2 × 10^17^ cm^−3^. In addition, the R_on_ and V_br_ increase with the thickness of the p-type layer, L_p_ (see Figure 6b). The optimum V_br_ and R_on_ are achieved for L_p_ around 300~400 nm.

## 3. Conclusions

In this study, a novel staircase design of a vertical GaN transistor without regrowth is demonstrated. The variable gate dielectric thickness along the channel helps to achieve a V_th_ of 6.6 V. A comparative study has been made with respect to traditional straight channel trench and multi-stage channel structures. The current density and the electric field profile in the channel of the staircase design show a superior control over channel carrier density compared to other two type of design. By adjusting the layer thickness and doping concentration, a specific device performance is accomplished, which provides adequate design flexibility for a wide range of applications. The stronger off-state effective channel resistance of the staircase structure leads to a high V_br_ of up to 2.12 kV and a R_on_ of 4.21 mΩ∙cm^2^ at a L_drift_ of 20 µm. For a balanced performance between R_on_ and V_br_, a V_br_ of 1.52 kV and a R_on_ of 2.61 mΩ∙cm^2^ can also be achieved at L_drift_ of 12 µm. The high breakdown voltage with low on-resistance suggests that the devices have the potential for high-voltage applications.

## Data Availability

The data presented in this study are available on request from the corresponding author.
